# Public Health Nurses’ Experiences in Preventing Obesity in Preschool Children. A Vignette Study From Child Health Clinics in Norway

**DOI:** 10.1177/23333936241274061

**Published:** 2024-09-20

**Authors:** Marte Cecilie Grøsvik Vinsjevik, Kirsten Gudbjørg Øen

**Affiliations:** 1 Stavanger Municipality, Stavanger, Norway; 2 Department of Public Health, University of Stavanger, Faculty of Health, Stavanger, Norway

**Keywords:** childhood obesity, prevention, public health nurse, vignettes, qualitative, Norway, Fedme hos barn, forebygging, helsesykepleier, vignetter, kvalitativ, Norge

## Abstract

This study explored the experiences of public health nurses (PHN) in preventing obesity in preschool children and following national guidelines. We used a descriptive qualitative design with an online open-ended questionnaire based on vignettes describing fictitious practical situations during the follow-up of children presenting with overweight issues in child health clinics. Following qualitative content analysis, we identified one main theme: Strong but complex feelings of responsibility toward supporting parents in preventing obesity in preschool children and adhering to the guidelines. We also identified four subthemes: (a) Difficulties in deciding if the child’s weight is a concern. (b) Challenges with meeting children’s healthcare needs when weight is a concern. (c) Approaches for engagement and caring weight talks with parents. (d) The need for resources and interdisciplinary collaboration. PHNs suggested improvements for preventing childhood obesity and called for municipal prioritizing of organizational and personnel-related resources to ensure PHNs can efficiently follow national guidelines.

## Abstrakt

Denne studien utforsket erfaringene til helsesykepleiere med å forebygge fedme hos førskolebarn og følge de nasjonale retningslinjene. Det ble brukt et beskrivende kvalitativt design med et nettbasert spørreskjema med åpne spørsmål basert på vignetter som beskriver fiktive praktiske situasjoner under oppfølging av barn med overvekt i helsestasjoner. Etter kvalitativ innholdsanalyse identifiserte vi ett hovedtema: Sterk, men kompleks ansvarsfølelse for å støtte foreldre i å forebygge overvekt hos førskolebarn og å følge retningslinjene. Vi identifiserte også fire undertemaer: (a) Vanskeligheter med å avgjøre om barnets vekt er bekymringsfull. (b) Utfordringer med å møte barns behov for helsetjeneste når vekt er et problem. (c) Engasjement og omsorgsfulle tilnærminger ved samtaler med foreldre om vekt. (d) Behov for ressurser og tverrfaglig samarbeid. Helsesykepleierne foreslo forbedringer for å forebygge overvekt hos barn og etterspurte kommunal prioritering av organisatoriske og personalrelaterte ressurser for å sikre at helsesykepleierne kan følge de nasjonale retningslinjene på effektiv måte.

## Introduction

The World Health Organization (2024) estimated that 37 million children under the age of 5 years were overweight worldwide in 2022. Obesity is a serious public health threat affecting many children in Europe (Spinelli et al., 2019). In Norway, data collected between November 2003 to December 2006 indicated the prevalence of overweight and obesity was 12.7% in the lowest age group (children aged 2–5 years) and 13.5% in a total sample of children ages 2–19 years, with obesity accounting for 2.3% of this figure (Juliusson et al., 2010). The most recent information shows a sample of 773 children from five Norwegian health centers reported a prevalence of 10.8% of overweight and obesity among children aged 2–5 years in 2014 (Westergren et al., 2021). Being overweight is defined as a body mass index (BMI) > 25 kg/m^2^, obesity is defined as a BMI > 30 kg/m^2^, and severe obesity as BMI > 35 kg/m^2^. BMI for children is adjusted for sex and age (Cole et al., 2000) and is called Iso-BMI.

Obesity is a complex condition that can lead to serious health consequences for a child—as well as later in adulthood—and contributes to numerous chronic diseases, including cancers, diabetes, metabolic syndrome, and cardiovascular diseases (Safaei et al., 2021). Further, children with obesity experience psychosocial problems, such as bullying, anxiety, depression (Şahin & Kırlı, 2021), and stigma (Haqq et al., 2021). Stigma contributes to behaviors, such as binge eating, social isolation, avoidance of healthcare services, decreased physical activity, and increased weight gain, which worsens obesity and creates additional barriers to healthy behavioral change (Pont et al., 2017).

Given the serious effect obesity can have on education, health, social care, and economic systems, public health experts suggest using a variety of approaches from early prevention to treatment of overweight and obesity (Spinelli et al., 2019). Primary prevention is essential to reduce obesity incidence, as practitioners stress it is easier to encourage the adoption of healthy eating habits than to intervene with diets in children who already have overweight issues (Nittari et al., 2019). Although preventing obesity requires steps to be taken at a young age and the involvement of parents, evidence concerning the effectiveness of preventive family interventions is limited (Landgren et al., 2020; Nordlund et al., 2022).

For parents to be promising change agents, they must first recognize that their child has overweight (Faith et al., 2012). Studies have shown that parents often misperceive their children’s weight and growth development and may not realize their children, especially young children, have overweight or obesity (Hudson et al., 2012; McKee et al., 2016; Toftemo et al., 2013). According to McKee et al. (2016), parents who misperceive their child’s weight are more likely to have overweight children. Moreover, parents who have overweight may not accept concerns about weight regarding their child, and as a result healthcare professionals can find these parents challenging to work with (Bradbury et al., 2018).

Although parents desire to be informed of issues concerning their child’s weight (Ames et al., 2020), parents of children younger than 4 years old are less prepared to hear about their child being overweight and have resisted help from public health nurses (Hanssen-Bauer & Knutsen, 2017). Moreover, parents might experience conversations with nurses negatively, as they may provoke feelings of failure and regret, or beliefs they are powerless to change the situation due to inadequate information (Eli et al., 2022). Parents can also experience such conversations as criticisms of their child and their parenting, leading to guilt and shame having a child with overweight or obesity (Hanssen-Bauer & Knutsen, 2017; Toftemo et al., 2013). Furthermore, parents may have concerns that focusing on their child’s weight could foster a negative body image (Eli et al., 2022). Thus, parents may wish to discuss their child’s weight development with a PHN when the child is not in the room (Hanssen-Bauer & Knutsen, 2017; Toftemo et al., 2013). During weight conversations, parents may be unsure of how to manage their child’s weight despite the child being identified with overweight or obesity (Hardy et al., 2019).

However, healthcare providers face several challenges when collaborating with parents to prevent obesity in children. PHNs and other healthcare professionals reported challenges related to their lack of knowledge and perceived competence and fears of parents’ reactions, risking their relationships with the parents, or harming the child (Bradbury et al., 2018; Helseth et al., 2017; Sjunnestrand et al., 2019). Moreover, PHNs highlighted a lack of time and resources for conducting meaningful conversations with parents (Bradbury et al., 2018; Davidson et al., 2018; Helseth et al., 2017; Nordstrand et al., 2016).

## Background

Norwegian national guidelines for preventing overweight and obesity recommend actions specifically targeting Iso-BMI categories (The Norwegian Directorate of Health, 2010). At Level 1, the municipality has a responsibility to encourage people to make healthy choices. At Level 2, PHNs are expected to intervene if the child’s Iso-BMI is between 25 and 29.9. At this level, the PHN should engage parents in a conversation about their child’s weight development and provide advice on nutrition, physical activity, risk factors, and preventative measures against overweight and obesity (The Norwegian Directorate of Health, 2010). At Level 3, the guidelines outline more resource-demanding interventions for children with Iso-BMIs between 30 and 34.9. If the child has an Iso-BMI > 35, a coordinator must then be appointed to ensure interdisciplinary collaboration, including the child welfare service, to provide parental guidance. Through the child health clinic (CHC) program, Norwegian children are regularly measured, often before the age of 2 years; thereafter, at the ages of 4, and the school health service measures children from the ages of 5 years (The Norwegian Directorate of Health, 2017).

The Norwegian national guidelines emphasize that motivational interviews (MIs) should be used in conversations with parents (The Norwegian Directorate of Health, 2010). MIs are a communication tool that acknowledges the other person’s self-esteem, promotes behavior change, provides nonjudgmental information, and elicits the patient’s understanding (W. R. Miller & Rollnick, 2013). Compared to usual care, MIs have positively affected parental influence over young children’s anthropometric measures (Suire et al., 2020). Furthermore, MIs delivered by registered dietitians have resulted in statistically significant reductions in BMI percentiles in children with obesity (Resnicow et al., 2015). A systematic review investigating current best practices for diagnosing and treating childhood obesity found that the most effective interventions used the MI technique and targeted families rather than children (Ahmed et al., 2023).

Previous studies from Norway have reported that PHNs experienced difficulties engaging with parents and following national guidelines for preventing overweight and obesity in children (Helseth et al., 2017; Nordstrand et al., 2016). PHNs described adopting attitudes toward the follow-up of children with obesity when adhering to the national guidelines, which range from being structured and pragmatic to critical (Nordstrand et al., 2016). Furthermore, school nurses reported that following the guidelines meant they were often assigned new tasks and responsibilities without sufficient training (Helseth et al., 2017).

Norway has a public, free-of-charge follow-up service for all children. PHNs at public CHCs follow childhood growth and development and play an essential role in guiding and teaching healthy habits in children and mapping children’s growth development in line with national guidelines. PHNs working at CHCs are key professionals tasked with discovering children at risk for obesity and acting. A Norwegian focus group study explored the experiences of PHNs working in CHCs with the “Step 1 Module,” which provides guidance for mapping, family guidance and counseling of healthy habits, and collaboration between PHNs and parents (Westergren et al., 2021). Overall, PHNs perceived that the module provided new and useful tools for addressing overweight and obesity among preschool children. However, PHNs described such work is intricate and the collaboration with families as “moving upstream in a river” because overweight and obesity may be one symptom of many complex challenges within families. PHNs considered the collaboration with specialist healthcare staff necessary for delivering this overweight and obesity prevention module (Westergren et al., 2021).

### Aim and Research Questions

This study was part of a larger cooperating project, Healthy Future, aimed at developing improved practices for PHNs to support parents of children at risk of developing obesity. Since little is known about how Norwegian PHNs experience working with parents to prevent childhood obesity, especially those with young children, this study focused on the experiences and practices of PHNs working at CHCs for preschool children. Overall, the knowledge gained from this research will help improve practices at CHCs to better meet the needs of parents. Through this study, we aimed to answer the following questions: (a) How do PHNs experience assessing and identifying overweight and obesity in young children, communicating these results to parents, and following national guidelines? (b) How can the employer and the management of CHCs support PHNs in their work of preventing overweight and obesity in children?

## Methods

We used a qualitative descriptive design for this study. Qualitative description research lies within the naturalistic approach, which seeks to understand a phenomenon through the meanings participants ascribe to it in their natural context (Bradshaw et al., 2017). Such research strives for an in-depth understanding of human phenomena first through literal descriptions (Sandelowski, 2010) and then through analysis and interpretation of meaning people ascribe to events (Bradshaw et al., 2017).

To begin, we constructed vignettes about hypothetical situations and persons from the field of prevention of obesity in children. Vignettes are brief descriptions of events or situations (fictitious or actual) to which respondents are asked to react and provide information about how they would handle the situation as described (Polit & Beck, 2017). Vignettes are used in qualitative and quantitative research to gain information about participants’ beliefs (Gourlay et al., 2014). As PHNs have reported difficulties and barriers to talking with parents about overweight in children (Bradbury et al., 2018; Helseth et al., 2017; Nordstrand et al., 2016; Westergren et al., 2021), we used vignettes so that PHNs could respond without feeling the discussion was too personal. According to Barter and Renolds (1999), vignettes are a less personal and, therefore, less threatening way of exploring sensitive topics that allow actions to be explored in context, clarification of judgments, and participants to define the situation on their terms. Vignettes can be pictures, texts, narratives, or short stories that present hypothetical case situations and are especially valuable for exploring perceptions, attitudes, and behaviors (Hughes & Huby, 2002).

We conducted online data collection as a good method to meet the requirements of social distancing and guarantee that all respondents received the same questions based on specific, practical situations to which they could relate. According to O’Cathain and Thomas (2004), open-ended questions allow participants to share their opinions and experiences of the theme and research questions. The second author developed the vignettes and open-ended questions based on her experience supervising nurses and midwives during further education in obesity prevention and her practical work with families with children with overweight or obesity. In particular, she was influenced by meetings with PHNs who conveyed the difficulties they experienced in implementing prevention programs and how negative media reports criticizing the standards of weighing and measuring and informing parents that their child had obesity made them question their effectiveness. We have been aware that the researchers’ preconceptions are important and might influence all phases of the research process. To limit researcher bias and encourage reflexivity, we discussed the vignettes and interview questions together and with colleagues to ensure they represented a variety of topics and situations. Discussions with and feedback from colleagues were important to stay open-minded and reflexive throughout the research process.

The vignettes presented in the online questionnaire contained three close-to-practice cases describing fictitious PHNs’ consultations with the parents of a child presenting with overweight or obesity. The fourth case describes a fictitious staff meeting to explore the nurses’ views about their employer’s responsibility to manage childhood obesity.

### Presentation of the Vignettes and Questionnaire

The first vignette described a case about a 2-year-old child’s weight and growth assessment:

*Today, Markus and his mother are visiting the child health clinic for his 2-year consultation. You have been following the family and are aware that Markus’s father and his 5-year-old brother have an overweight problem. As Markus’ PHN, you are going to assess Markus’ growth.*

Q1: As a PHN, how would you proceed when identifying and assessing whether Markus is overweight or obese? Explain and describe in your own words how you would proceed.

Q2: What would you emphasize in your assessment if Markus is identified as overweight or obese?

The second vignette was about communicating weight results to parents. The respondents were asked to share their experiences in holding conversations with parents in situations similar to this case:

*Today, Johanne is visiting the child health clinic for her 4-year consultation. You have measured Johannes’ height and weight and found that she has an Iso-BMI of 31. How would you present the result to Johannes’ mother and begin a conversation with her about what the result might mean?*

Q3: Can you describe your experiences holding conversations with parents in situations like this?

The third vignette described a case of a child who was overweight at the previous consultation and new weight measurements indicate the child is now classified as obese. The respondents were asked to consider how they would address the situation knowing that the mother canceled the last planned follow-up appointment for weight control:

*Today, Linn is at your office for a preschool consultation; she is 6 years old. Her record from the 4-year check-up showed that she had an Iso-BMI of 29. After weighing and measuring today, her Iso-BMI was calculated as 33. It is noted in the record that the mother cancelled the offer for a follow-up talk about weight development after the 4-year check-up and did not attend the appointment at that time.*

Q4: Based on the information you have, describe how you would deal with the situation now that the weight trend has worsened.

Q5: What specifically would you do?

The fourth vignette refers to an arranged staff meeting for all employees at a CHC in the municipality where the lead PHN (representing the municipalities’ management of childhood obesity) wants them to discuss what it is like for them to follow the national guidelines when handling overweight and obesity in children. The lead PHN wants to ensure the municipality is providing the best possible health services to children who are overweight and obese and their families and asks the employees to be as open and honest as possible.

Q6: Can you tell us about your experiences with following the national guidelines for the follow-up of children with overweight and obesity and carrying out what is expected of you as a PHN based on the guidelines and meeting the family’s needs?

Q7: How can the employer make working with overweight and obesity in children easier for you as a PHN?

The initial section of the questionnaire included questions about age, education, work experience, and professional degree. We conducted a pilot test of the questionnaire with an experienced PHN, after which we made several adjustments beginning recruitment. We estimated that answering the questionnaire would take approximately 20 min.

### Recruiting Participants, Settings, and Data Collection

The study was conducted during the lockdown due to the COVID-19 pandemic. We contacted PHNs via email. To begin, we sent emails to the management of CHCs in municipalities that had participated in the larger project. The lead PHN forwarded the emails with the voluntary survey to the PHNs in CHCs in their municipality on October 25, 2021. The inclusion criteria were registered PHNs with a minimum of 1 year of experience working with obesity in young children. However, due to a low number of respondents, we sent an email reminder to the managers after 14 days, which only resulted in a few responses. Therefore, we contacted the managers of CHCs from 10 other municipalities on November 23, 2021. The total recruiting process lasted from October 8, 2021, until January 10, 2022, when we received 20 surveys in the SurveyXact (Ramboll, Oslo, Norway) database. Seven surveys were not completed and excluded. Of the 13 completed surveys, two nurses who were not registered PHNs were included because they had many years of experience working with children with obesity, had relevant education, and provided valuable information to the research questions.

### Data Analysis

Qualitative content analysis comprises descriptions of the manifest content, close to the text, as well as interpretations of the latent content, distant from the text but still close to the participants’ lived experiences (Graneheim et al., 2017). The latent content is the interpretation of the underlying meaning or the “red thread” between the lines in the text (Graneheim & Lundman, 2004). The descriptions and interpretations can be seen as emanating from phenomenological and hermeneutic approaches to the objects of the study (Graneheim et al., 2017).

PHNs provided answers to the open-ended questions in text form, which both authors analyzed using the qualitative content analysis process outlined by Graneheim and Lundman (2004). This method focuses on the respondents’ experiences, reflections, context, and variations and enables several possible and valid interpretations using a systematic approach (Graneheim & Lundman, 2004). Most of the respondents had written full and thorough answers, which made it possible for us to analyze both manifest and latent content.

The different phases of the analysis followed the process described by Graneheim and Lundman (2004; Graneheim et al., 2017). First, we read the text several times to gain an overall understanding of the material. We then identified the meaning units in the text, which consisted of phrases or sentences related to our research questions. Lastly, we condensed and coded the meaning units.

Next, we considered the similarities and differences among codes and grouped content accordingly into categories and subcategories, relying on the manifest content of the participants’ experiences expressed in the text. We then searched for underlying meanings in the answers, which required a deeper interpretation of the text. During this process, we identified four subthemes from the latent meanings of the respondents’ experiences, which we interpreted as branches of a main theme. This main theme was identified after connecting latent meanings to a common recurring theme, according to Graneheim and Lundman (2004). Throughout the analysis process, the authors discussed the themes until consensus was reached (Table 1).

**Table 1.** Example of the Analysis From Meaning Unit to Subtheme (d) and the Main Theme.

Meaning unit	Condensed meaning	Underlying meaning	Sub-theme (d)	Theme
*“The families have far too little service for follow-ups over time.”*	Too little follow-up	Families have needs for follow-up that are not met	The need for resources and interdisciplinary collaboration	Strong but complex feelings of responsibility toward supporting parents in preventing obesity in preschool children and adhering to the guidelines
*“Change takes time, especially lifestyle changes.”*	Follow-up and lifestyle changes take time	A time-consuming task that helps with lifestyle change		
*“It should be an offer . . . where the family receives guidance in the afternoon, as an entire family.”*	Offer the entire family guidance in the afternoon	Suggestion for a more user-friendly follow-up for the entire family		
*“The GP [general practitioner] must also examine the child and there may be referrals/examinations, which entail a trip to the obesity clinic.”*	General practitioners must examine and refer children to obesity clinics	General practitioners must do their duty and follow up and make referrals to specialists		
*“I have heard of families who have come there and experienced this journey as tiring and therefore cut it short.”*	Families experience follow-ups in the hospital as tiring and soon stop	Follow-ups in the hospital are demanding for families, and they quit		
*“I have contributed to a lot of unrest in the family, which, in turn, can create a bad sense of self in the child and, in the long run, also in parents in terms of their parenting.”*	Contribution to unrest and bad feelings for the child and the parents	Uncertainty about whether follow-ups in the hospital are doing more harm than good for the child and the family		
*“Should have regular meetings with a manager or other contact person for supervision.”*	Regular meetings with a manager for supervision are desired	Supervision from managers is sought after		
*“I should get good guidance in this work, especially regarding dialogue and care for parents, who will then be secure in parenthood.”*	Need good dialogue and guidance to help the parents be secure in their parenthood	Sought after communication guidance to care for parents and help them feel secure about their parenthood		
*“Coordinate the different guidelines within the different professional fields . . . work with common attitudes and knowledge about obesity.”*	Need for coordination of different professional fields and work for a common understanding of obesity	Sought for better coordination of and work for a common understanding on how to follow up with children with obesity		
*“Have all professional groups available for use regarding obesity in children.”*	Need for available professional groups	Sought for a multi-professional group to refer to		

### Research Ethics

All participants received information about their rights in an information sheet, which we sent in the initial email together with the vignettes. We safeguarded participants’ privacy through the data program SurveyXact, without any possibility of tracking the IP address of the respondents. Hence, respondents did not have the opportunity to withdraw their answers. No approval from the Norwegian ethics committee or the Norwegian Centre for Research Data was needed. Our research does not involve any personal data concerning the physical or mental health of a natural person, including the provision of health services (The Norwegian Health Research Act, Chapter 1, §4d). Our study was based on fictive cases and explored what the nurses would do in such cases. This study followed the Helsinki Declaration of 2013 (World Medical Association, 2013). The study was submitted to the Norwegian Regional Ethics Committee West and assessed as not subject to application (number 655787).

## Results

The participants were PHNs working at CHCs in municipalities in southern Norway. All 13 PHNs were highly educated and experienced with following up children with overweight. The respondents age varied between 27 and 64 years. 11 PHNs had master’s/further education in public health nursing (PHN), 2 had bachelor’s in nursing (+ post graduate course). Work experience varied between 5 and 11+ years.

The length of the respondents’ writings varied, with longer stories when PHNs experienced challenges in their work. The PHNs’ answers were interpreted and described into one main theme: Strong but complex feelings of responsibility toward supporting parents in preventing obesity in preschool children and adhering to the guidelines.

PHNs described perceiving overweight and obesity problems in children as a health threat and a societal problem. Respondents were concerned about the significant effect obesity has on children’s health and compared obesity with neglect and disability, seriously affecting the child’s future life opportunities. PHNs were aware that they have a central role in both preventing and following up children with overweight and obesity, which was clearly stated in the Norwegian national guidelines. Respondents wanted to be sure that they judged the weight problem in a child correctly and, therefore, used several sources for information before concluding whether to alert the parents. Overall, PHNs strived to reach out to the parents in a caring way when overweight or obesity was identified. However, some PHNs found national guidelines challenging, overly comprehensive, and resource-intensive and experienced conflicts between following the guidelines and safeguarding children and their families. These conflicts were due to difficulties in trusting the measurement curves when communicating overweight to parents who were unaware that their child was overweight. PHNs stated it was difficult for them to give messages that could create negative feelings in the parents. PHNs stressed that families with a child with overweight should be offered better follow-up. The respondents described that they lacked the necessary skills in communication and behavior change methods, time resources, multidisciplinary collaboration opportunities, and organizational and managemental support. PHNs are included in the guidelines as a professional group with a clear responsibility for following up with children with overweight and obesity even though they felt that they lacked the necessary training and resources to do so, which led to an internal conflict in PHNs. To increase the quality of their follow-up of children with overweight or obesity, PHNs stated that the municipality should prioritize the necessary resources and facilitate a local multidisciplinary team for family referrals.

We also identified four subthemes: (a) Difficulties in deciding if the child’s weight is a concern. (b) Challenges with meeting children’s healthcare needs when weight is a concern. (c) Approaches for engagement and caring weight talks with parents. (d) The need for resources and interdisciplinary collaboration.

### Subtheme a: Difficulties in Deciding If the Child’s Weight Is a Concern

Even though PHNs responses to vignettes indicated they understood how to conduct comprehensive assessments and their use of several sources to determine the severity of the child’s weight situation, it was clear in their responses they had difficulty in deciding if a child’s weight should be a concern.

PHNs focused on conducting a comprehensive assessment and used several sources to determine the severity of the child’s weight situation. At each appointment, PHNs stated they measured the child’s height and weight and mapped the results on the growth curve to identify a steady climb or a fast-growing tendency. The PHNs believed the growth curves were important for assessing and identifying overweight children. As PHN 7 explained, “*It is important for me to look to the growth curve when I shall assess the child’s weight situation.”* However, some PHNs doubted the trustworthiness of the growth curves and explained that it was challenging to have confidence that a specific child should be categorized in a specific overweight category, especially small children assessed with obesity. PHN 5 wrote, “*There can sometimes be a mismatch between curves, Iso-BMI, and the impression I have of the child*.”

Respondents considered the national guidelines too rigid and diffused because the interventions described in the guidelines were difficult to implement due to a lack of resources, high workload, and unsuccessful attempts to motivate parents. Notably, PHNs stated that measurements and growth charts alone provided little information about a child’s health and development. Some also wrote they considered a child’s physical appearance, musculature, motor skills, comparisons with earlier measurements, and family disposition. PHNs 10 wrote, “*I will study the growth charts and assess the child’s physical appearance.*” Similarly, PHN 13 wrote, “*I would look at his Iso-BMI but also consider musculature*.” PHNs stated they also attempt to identify the underlying causes of the child’s obesity by mapping risk factors in daily life activities and habits as well as inherited risk factors. PHNs wrote they would consider the child’s motor skills, activity levels, and eating and drinking habits within the family when they found an issue with overweight or obesity.

Another tool PHNs used to assess the child’s weight category included two-way communication with the parents. PHN 13 stated. “*To consider the child’s weight situation, it is important to establish a good relationship with parents. A good relationship is important for my evaluation of the child’s situation and to be able to give good overall feedback.*” PHNs emphasized the parents’ role in helping them understand a child’s weight situation and that the parents’ views and ideas about their child’s weight development should be valued. For instance, concerning Vignette 1, PHNs stated they would ask Markus’ father what he thought about the changes in Markus’s growth. Together with Markus’ father, most PHNs wrote they would conduct a comprehensive mapping of Markus’ dietary history. PHNs stated a broad screening was necessary in Cases 2 and 3, where obesity was identified. In addition to mapping diets, several respondents wrote they wanted to map sleep habits, physical activity, and well-being both at home and school.

To address difficulties in deciding if the child’s weight was a concern, participants frequently suggested that collaboration with other specialists for the assessment of children’s weight situation was important. Many PHNs described general practitioners (GPs) and physiotherapists as playing important roles in the assessment of a child’s weight situation. PHNs wrote they wanted, if possible, to refer to a nutritionist, but stated that not all municipalities had this professional resource available. PHNs stated that they provided information to parents about other professional groups and asked for consent to start working together with their GP. PHNs considered it important to discuss Markus’s weight with his GP and stated that, based on the guidelines, the child needed medical supervision to rule out underlying disease as a cause of the overweight and obesity.

### Subtheme b: Challenges with Meeting Children’s Healthcare Needs When Weight Is a Concern

Although the PHNs reported that they were concerned about the children’s health in cases of obesity, they experienced falling short and providing insufficient health care due to a lack of resources and insufficient cooperation with other agencies and with the parents.

The respondents reported they mostly could deliver follow-ups according to the guidelines, but their effectiveness depended on others’ motivation. PHN 7 wrote:

*I try to follow the guidelines. Mostly, it goes well when the families are motivated for change. However, I have experienced some resistance from GPs regarding follow-up, they do not see the need; they think it is a parental responsibility. Some parents do not see a need for change; then it becomes difficult when you have to spend a lot of time motivating and getting in position to help.*

PHNs felt the guidelines provided helpful interventions if obesity was identified, but with more severe obesity, PHNs experienced increased concern for the child’s health. Respondents described that it was challenging to propose the parents seek support and help from child protection. As PHN 2 explained, “*When do we talk about the obesity problem in relation to neglect? There are far too many children and young people who are deprived of their opportunities at an early age due to the wrong lifestyle.*”

PHN 3 wrote:

*The guidelines are good on obesity. But connecting child protection and forming a responsible group is further down the line.*

The respondents shared a desire to help families with children diagnosed with obesity. In Linn’s case, several PHNs wrote they would arrange additional follow-ups for weight control, counseling, and guidance, send referrals to a doctor, and establish a responsibility group. In the first case (Markus), the father and one brother were described as overweight. Some respondents pointed out that this situation would require behavioral changes for the entire family to prevent further development of overweight and obesity, and that children rely on their parents’ understanding of diet and underscored that parents should act as important role models to reverse a child’s increased weight. PHNs wrote they would give advice and guidance about healthy eating habits, foods with hidden calories, especially sweet drinks, and advice on dental health. PHNs also stated they emphasized the social interaction around meals with parents and proposed that follow-up interviews based on dietary tools should be used. PHN 13 wrote:

*I would also talk about the social aspect of the meal, about the fact that children at this age will often help to set the table, they will help and feel significant. In the survey, as first aid, I would have gone into sugar in the diet.*

However, overall, PHNs found it difficult to bring about change as parents often provided excuses for unsuccessful dietary changes.

Concerning collaboration, PHNs stated they would initiate cooperation with others and establish a responsibility group for coordinated follow-up service if a child was identified with overweight or obesity. PHN 12 explained how she would have conducted the follow-up of Markus:

*I would have considered his Iso-BMI result. If he is in a danger zone, I refer him to a GP and establish a group of responsibilities or, if his degree of overweight is lower, I would start with a follow-up at the child health clinic and make a follow-up plan from here.*

Collaboration with a physiotherapist was cited by several respondents to thoroughly map motor development and skills when obesity was identified. PHN 12 wrote that she assessed Markus’ situation as follows:

*With Iso-BMI 31, the national guidelines tell me the family should be followed up by a public health nurse. Also, the family should be connected to resource persons in the child’s environment, possibly a physiotherapist, and others in the family or kindergarten, etc.*

PHNs experienced overweight and obesity problems in children as a health threat and a societal problem. However, PHNs stated that following the guidelines and involving child protection services when a child had obesity was difficult. PHN 2 stated, “*But connecting child protection services and forming a responsibility group is further down the road. Then you should have achieved a good helping relationship with the family. Most parents do not want help from the child protection services.*”

A significant finding was that the respondents experienced the healthcare service for children with overweight and obesity to be insufficient. PHN 10 stated, “*Just weighing and measuring height should not be the only intervention we offer.*” PHN 4 wrote, “*Family members have far too little support for follow-ups over time. Change takes time, especially lifestyle change. It should be offered . . . where the family receives guidance in the afternoon, as a family.*”

### Subtheme c: Approaches for Engagement and Caring Weight Talks With Parents

PHNs believed overweight was a difficult topic for parents, which PHNs wanted to acknowledge in conversations. All PHNs stated they were concerned about communicating issues of overweight and obesity using a caring and careful approach focused on the parents’ perspectives. They wrote they wanted to spend time on dialogue and first building relationships with the children and parents as a strategic and caring approach to a sensitive topic. PHNs stated that good dialogue between parents and PHNs was the foundation for greater trust and cooperation. PHN 1 wrote:

*By first “seeing” and acknowledging the child and the parent and first talking about what the parents wanted to bring up. Then, a good relationship between them would occur and would promote a good start on communicating about the child’s overweight.*

PHNs wrote they focused on positive health benefits, which they meant served as an important basis for further follow-ups. PHN 12 would explain to Linn’s mother that *“the goal is for her to grow into a better [growth] curve. Emphasise a positive focus, positive health benefits and well-being as positive consequences of better weight development.*”

The PHNs stated they provide information and support through dialogue and seek out resources to support the entire family if they are open to change. PHNs wrote that it was important that parents did not feel defensive, which would create a barrier to their further cooperation. Respondent 9 shared her experiences regarding the case of Linn, where weight development worsened after the prior consultation:

*I find that the relationship between parents and public health nurses is very important to be able to get in a position to help. I always focus on parents not feeling that they must defend themselves and that I raise the issue for physical, mental, and social reasons and for the child’s health purposes.*

Our results indicated that the presence of overweight family members affected how some PHNs would approach them and what measures and advice they would provide. A cautious approach to the topic was emphasized in a description in which the father and brother were also overweight. Several respondents wrote they assessed the procedure according to the family’s situation and as part of a careful dissemination of the topic. PHN 11 wrote, “*The fact that the family member is also overweight means a lot for how I can approach the family with advice, guidance and motivation for behaviour change.*” PHNs stated conversations were most beneficial when using a careful approach and prejudices were set aside.

PHNs described situations when parents were surprised by information about their child’s weight and did not understand that their child had overweight or obesity. According to PHN 1, “*many parents convey that they cannot understand it, as they eat healthily and most 4-year-olds are active [in the parents’ assessment]. I am, therefore, reluctant to give any good advice at the first meeting*.” When parents were aware of their child’s weight development, the PHNs found it easier to have a conversation, as they were more receptive to discussion, guidance, and further cooperation.

Several respondents stated they found it ethically challenging to talk about overweight with the child in the room. When the child’s weight was raised in the consultation, several respondents wrote they considered how the child was included in the conversation. PHNs stated they were conscious of their choice of words when the child was in the room and would offer parents a private conversation with time for a thorough assessment. PHN 9 wrote:

*In situations where the child is 4 years or older, I tell the mother/father that I want a parent conversation about the growth measurements. In my experience, a first conversation with the child present rarely becomes a good or useful talk . . . parents agree that this is an adult talk.*

Concerning Case 3, when a parent decided to cancel a follow-up appointment, PHNs expressed various opinions. Some PHNs regarded it as her responsibility to follow up with a new appointment, whereas others wondered whether the parents found it difficult to attend that specific appointment. Some respondents stated they chose not to comment on the mother’s cancellation of the appointment to maintain trust in the relationship and focused instead on helping and conveying hope. Other respondents wrote they chose to ask the mother about the canceled appointment as part of a responsible conversation to help the mother understand the situation.

### Subtheme d: The Need for Resources and Interdisciplinary Collaboration

The respondents reported that they experienced the inadequate healthcare they offered to children with obesity could be improved if the management, the municipality, and the health authorities prioritized obesity prevention. The PHNs wrote that the municipality should take the responsibility of offering healthcare for families with overweight or obese children, and that lack of sufficient service felt challenging for the PHNs. PHN 7 explained, “*The municipalities should have been required to have close follow-up in relation to good food courses, guidance from the healthcare provider in relation to the dietary tool, physiotherapists who took care of mapping and activity.”* PHN 3 wrote, “*It is challenging that we have so little to offer these families.*”

The PHNs described obstacles they experienced in following up with the children. A prominent finding was the lack of resources. Specifically, PHNs struggled to find the time to prioritize the work needed to follow up with children with overweight and obesity, as this task only represents part of their duties. However, resource challenges went beyond follow-ups, as PHNs also perceived the coordination of many professional partners as a resource requirement. PHN 9 wrote:

*But I always face resource challenges, that means that I never get to follow up closely enough. There are many to collaborate with. Many of the children may be entitled to a coordinator and an individual plan for help in the future.*

A robust finding in this study was the PHNs’ feelings of being alone when supporting parents in addressing overweight or obesity. Several PHNs expressed challenges linked to collaboration with GPs when different views on overweight and obesity problems emerged. PHNs wrote some GPs did not refer children with obesity to specialist healthcare services, despite a clear recommendation based on the PHNs’ measurements and according to the national guidelines for overweight and obesity in children. A lack of routine and optimal interdisciplinary cooperation and communication between various professionals were seen as barriers that resulted in parents not receiving adequate follow-up. Moreover, the lack of cooperation with other agencies made it difficult for PHNs to work according to national guidelines. In cases when interventions were implemented by the specialist healthcare service, PHNs stated they often failed to send information back to the PHNs, which hindered further follow-ups in their municipality.

The respondents believe that employers are responsible for healthcare in cases of overweight and obesity in children and shared several suggestions regarding measures that management could implement to ensure work is easier for PHNs and, at the same time, deliver better healthcare for children and their families. Employers could contribute by prioritizing more resources, such as more time for each consultation, especially more time for follow-up meetings and extra consultations with parents, time for discussions and interdisciplinary responsibility groups, and facilitating better communication between the disciplines. PHNs thought employers should recognize the importance of follow-ups and be educated on the topic of obesity in children. PHNs also expressed a need for support and supervision talks with their managers. PHNs wanted courses in MIs and skills development on how to prevent obesity in children. PHNs felt increasing their level of professional competence in conversational skills would provide a sense of security during difficult conversations with families.

Discussing the topic with colleagues was also interpreted as beneficial social support for PHNs’ health by increasing their safety when assessing overweight and obesity in children. However, respondents commented that some municipalities lacked groups of relevant and available specialists such as nutritionists, occupational therapists, and physiotherapists. The PHNs also believed that GPs should be informed about the content and requirements of interventions that follow national guidelines and that the management of primary healthcare in municipalities should ensure this.

Although PHNs perceived the organization of healthcare to be a social responsibility, the organization of healthcare for children with obesity in the specialist health service was identified as a barrier because families were often required to travel long distances for follow-ups. PHNs found it ethically challenging to follow the guidelines, as examinations, treatments, and traveling to specialist healthcare services contributed to family stress. Further, the intense focus on the child’s obesity could lead to poor self-esteem for parents and children. PHN 13 wrote:

*The GP must also examine the child, and there may be referrals/examinations that entail a trip to the obesity clinic, blood tests that are painful, and so on. I have heard of families who have gone there and experienced this journey as tiring and, therefore, cut it short . . . then I have contributed to a lot of unrest in the family that, in turn, can create a bad sense of self in the child and, in the long run, also in parents in their parenting.*

PHNs suggested moving obesity clinics to the municipalities and called for prioritizing healthcare resources in the municipality to prioritize reliable, complex, and comprehensive follow-ups of children with overweight and obesity. Participants stated it was the employers’ responsibility to oversee the use, management, and availability of professional resources. Respondents also proposed earmarking resources from the government for the treatment of children with overweight and obesity. PHNs stressed that local follow-ups would be more affordable for families to participate in consultations and would further provide opportunities for families to meet others in the same situation. Employers could encourage the municipality to prioritize a low threshold of healthcare for a fixed, interdisciplinary follow-up program.

## Discussion

The overarching theme for this discussion is PHNs’ experiences in assessing, preventing, and caring for children with overweight and obesity and how management and municipalities could improve the quality of healthcare for families with overweight children. The main finding in this study is that the PHNs experienced a strong responsibility to follow the national guidelines for follow-up of children with overweight and obesity despite several barriers. PHNs highlighted it was important to consider the accuracy of the child’s weight situation and that the guidelines were often too rigid or diffused. Participants also emphasized the importance of mapping, where PHNs used both their specialist knowledge and judgment. Overall, PHNs strived to create a good relationship with the parents and described taking several steps to communicate overweight to parents in a caring way. To offer better follow-up for families, the PHNs stressed that management and the municipality should prioritize more resources and ensure the available interdisciplinary collaboration partners. To reduce the stress on families, PHNs recommended that healthcare for children with overweight and obesity should be available locally.

In our study, PHNs felt they had the important responsibility of delivering healthcare services to children with overweight and obesity. However, they strive to support parents with lifestyle changes and finding the underlying causes of the child’s overweight.” PHNs ascribed to the use of a broad and holistic screening approach in line with the guidelines. Notably, PHNs did not mention considering medical causes, which may be because such causes are rare, and their mapping is the GPs’ responsibility.

Our results indicated that PHNs face many challenges when caring for families with children with overweight or obesity. The PHNs in this study recognize the importance of the national guidelines (The Norwegian Directorate of Health, 2010), are familiar with these guidelines, and are attempting to follow the guidelines. In response to vignettes, the PHNs in our study stated they used multiple sources to identify weight concerns among children. This finding contrasts with at least one Irish study that reported healthcare professionals did not conduct obesity-related clinical assessments among school-age children other than measuring height, weight, and BMI (Ferdous et al., 2023). Nevertheless, despite complying with the guidelines, concerns about the rigidity of the national guidelines raised concerns among the PHN in this study about the accuracy of their weight assessments as well as the value of some assessments, such as the growth charts. An earlier Norwegian study reported that PHNs questioned the body mass index cut-off of 25 Iso-BMI as the starting point for intervention (Helseth et al., 2017). Overall, PHNs in our study believe the growth charts alone provide little information about a child’s health, and that they would consult other professionals, discuss with parents, and use their judgment to determine the level of concern.

In our study, respondents stated that weight conversations with parents were challenging, as they are conscious of how difficult and vulnerable it could be for parents to hear that their child had obesity, and therefore emphasized using a nonjudgmental communication style, like PHNs in the study by Farnesi et al. (2012). Moreover, parents have also expressed a desire for such a communication style (Bentley et al., 2017). Our study showed PHNs prioritize a good relationship with parents to ensure they can help the family, a result found in other studies (Nygaard & Øen, 2024; Ray et al., 2022; Serban et al., 2021; Sjunnestrand et al., 2019). Other studies have also reported that parents believe trust leads to greater comfort with the provider and feelings of better quality of care (Bossick et al., 2017; McPherson et al., 2018).

PHNs in our study wanted to protect the child and did not want to bring up the overweight issue when the child was present, which is in line with Nygaard and Øen’s (2024) findings. Instead, our respondents would offer a new appointment for an adult conversation, like providers sampled by Toftemo et al. (2013). In the same vein, respondents in our study described being extra careful during their conversations if obesity was observed in parents or other family members, a consideration Edvardsson et al. (2009) also reported.

Another step our respondents experienced they would take in discussions with parents was to emphasize the health aspect of the weight condition. Research has found that parents of small children (aged 0–2 years) are strongly dedicated to their child’s health and that health risks were seen as a potential call to action (Canfell et al., 2022). However, focusing on the health risks should be approached with caution to avoid extreme actions by parents in response to receiving an at-risk result for their infant (Canfell et al., 2022).

The PHNs in our study also found it difficult to bring about change, as parents often provided “excuses” for unsuccessful dietary changes. Sjunnestrand et al. (2019) reported that parents sometimes lack awareness of their child’s obesity. Bradbury et al. (2018) also reported that healthcare professionals experienced difficulty promoting lifestyle changes when parents were unmotivated to change, lacked acceptance of the weight problem, or were overweight themselves. The perception by some PHNs that parents make “excuses” or are being unmotivated might have several other interpretations than just bringing problem responses. Other interpretations could be that parents are unsure about what to do and how to implement changes, and children may also have several eating issues and food preferences that add an extra layer of difficulty for the parents. Parents might be anxious about saying or doing the wrong thing when regulating children’s food intake (Fiskum et al., 2022). Such “excuses,” often arising from complex feelings, would trigger the PHNs’ curiosity, followed up by exploration with open questions to gain insights into the parent’s experiences and concern. Other of our informants stated that they would initially acknowledge the child and the parent and start the conversation by asking what the parents wanted to bring up, which is more in line with the spirit and strategy of motivational interviewing. Our respondents stated that they are aware that the Norwegian guidelines recommend using motivational interviewing to encourage parents to change their behavior, and PHNs would use this strategy “to roll with resistance” if parents proved to lack motivation to change, in line with suggestions by W. R. Miller and Rollnick (2013) who recommend meeting people with compassion, confirm their experience, and explore their further reflections by use of open questions. Earlier research has shown that when parents receive feedback on their children’s weight using motivational interviewing, they are more satisfied with the support they get from the healthcare provider (Ames et al., 2020). When nurses initiate conversations in a responsive, non-blaming way, inviting parents to reflect on their situation, parents felt supported and empowered (Eli et al., 2022).

In our study, PHNs differed in their opinions of how to act when parents ignored their child’s obesity (Vignette 3). According to the guidelines, in the case of obesity in the child, PHNs have a main responsibility for connecting with several specialist groups under the supervision of a coordinator (The Norwegian Directorate of Health, 2010). However, Westergren et al. (2021) reported that Norwegian PHNs were uncertain about the role they were supposed to play within the interdisciplinary cooperative approach between primary care, the special health service, and schools. Similarly, other researchers have also identified multi-disciplinary challenges and a lack of role clarity (Kelleher et al., 2017; Nordstrand et al., 2016).

The PHNs in this study feel the follow-up for families with a child with overweight is insufficient. The most prominent barrier we found in our research was structural, which aligns with the findings of another study (Taghizadeh et al., 2022). A lack of time, skills, and resources for follow-ups was prominent in our study. For PHNs to offer evidence based and systematic follow up of obesity in children, they need a significant amount of work hours, skills, and resources. For example, evidenced family-based behavioral treatment for childhood obesity includes approximately 26 sessions over a 24-month period (Epstein et al., 2023). This level of health care service is rare in Norwegian municipalities. Norwegian PHNs perceived that they have competence in health promotion and prevention work at the individual, group, community, and system level (Dahl et al., 2022). These findings may point to the need to find other solutions to how children with obesity are followed up within primary care services.

PHNs in our study felt isolated and experienced a lack of collaboration partners, such as GPs, to determine when a child with obesity should be referred to specialist healthcare services. Nygaard and Øen (2024) reported similar results but also that PHNs working in small municipalities reported good and close cooperation with GPs. In the present study, PHN mentioned that information was not routinely provided by the specialist healthcare service, which could hinder PHNs’ follow-up with families in the municipality and were seen as barriers that resulted in parents not receiving adequate follow-up. Our participants called for coordinators and individual plans to be offered to children with obesity and their families, and that the government should allocate earmarked funds for the prevention and follow-up of children with obesity in the municipality.

The PHNs in our study experienced concern for children’s future opportunities due to negative lifestyle habits and raised questions about when the child protection system should be involved in cases of obesity. However, it was difficult for PHNs to follow the guidelines referring children with obesity while most parents did not want help from the child protection system. Regber et al. have explored and described circumstances when health professionals would refer a child to the Social Service or consider foster care placement. These professionals concluded that the most obvious indication was in cases of parental inability to respond to the necessary lifestyle changes to support their child’s health and wellbeing. Another indication was several booked appointments being canceled and longstanding treatment not leading anywhere (Regber et al., 2018). Further, Regber et al. (2018) reported that a pediatric nurse felt that a positive effect of a notification to Social Services was to make the parents aware that they might need extra support to handle the situation, and that a limit had been reached and that society must protect the child in some way; however, a negative aspect was the risk that some families might break off contact with the health care provider. Nelson et al. explored staff perceptions of childhood obesity as a child protection issue. They concluded that the question of whether obesity alone is a child protection concern, is contested. Families who fail to recognize that child obesity is harmful to children and the failure of families to engage with support services was thought to potentially constitute neglect. When making judgments about child obesity and levels of harm, personal views about obesity and value judgments regarding parenting and children were as important, if not more so, than factual knowledge (Nelson et al., 2021). To our knowledge there is very limited research on use of child protection systems in cases of obesity in children to build on.

An important finding in our study was that PHNs believe health services for children with obesity are a social responsibility that both management and the municipality need to prioritize. Our respondents also called for more resources in terms of time, support, and staff training, which aligns with other studies (Bergström et al., 2020; Ray et al., 2022; Taghizadeh et al., 2022). An alternative solution to the challenges that emerge in this study could be to organize the follow-up in the primary healthcare service through multidisciplinary teams with a coordinator. Health services in the Netherlands have developed a Professional Integrated Care Model for local integrated care for children with overweight or obesity and their families (Halberstadt et al., 2023). The use of a coordinator in this project shows promising results. Parents’ experience so far shows major points of improvement concerning the intensity of the follow-up and collaboration (De Laat et al., 2022).

Our findings show a need for building professional capability, access to training, and referral services, which aligns with other studies (Mâsse et al., 2018; M. Miller et al., 2007; Ray et al., 2022). Evidence exists supporting the idea that primary healthcare providers can successfully prevent and treat childhood obesity by coordinating efforts within the primary care setting and linking obesity prevention and treatment resources within the community (Vine et al., 2013).

### Strengths, Limitations, and Future Directions

A strength of this study is the insight we provide into PHNs’ experiences of their duty when identifying children with overweight or obesity. A strength of the study was also our highly qualified and experienced respondents with insights into the phenomenon under study. As the Norwegian municipalities have different resources for obesity prevention and organize the work differently, it is an advantage that our results cover PHNs from several municipalities. PHNs are on the frontline of caring for families and their narratives of their work conditions provide ideas for preventing obesity in children and how to follow up based on the national guidelines; however, little data exists on PHN’s perspective on obesity prevention within Norwegian CHCs. Further, to our knowledge, clinical fictitious vignettes have not been used in other studies exploring the prevention of obesity in children. Vignettes represent an interesting and innovative method, which is also time-saving and easy to use. Vignettes gave another kind of data than if the PHNs had been interviewed about their experiences meeting real families in their daily work. Using vignettes, respondents could recognize themselves in concrete, practical situations, which may have led to more engagement and comprehensive answers to the questionnaire (Hughes & Huby, 2002; Sheringham et al., 2021). Moreover, PHNs could describe their experiences without risking privacy because the vignettes present fictitious families. However, we must acknowledge that our data are limited because they are based on the PHNs’ reflections and not their actions. Thus, we cannot know if respondents are answering based on what the guidelines say they should do rather than what they do or judge the care offered to families.

One step we took to increase the credibility of the study was using an online questionnaire, which included vignettes piloted by a PHN with experience in the phenomenon. Conducting a pilot was important to ensure the vignettes stimulated the informants to share their experiences and that the questions were understandable. This process also allowed us to make necessary corrections based on feedback.

Concerning the recruiting process, we found it difficult to recruit PHNs willing to participate. However, using an online data collection method meant we were able to recruit participants continuously until 13 PHNs had completed the questionnaire, which was a sufficient number to provide rich data and cover variation (Graneheim et al., 2017). The recruiting method has advantages and drawbacks. First, due to data anonymity, we could not directly send a reminder to potential participants to complete the survey, as this was left to managers of CHCs who forwarded the invitation email. Difficulties in accessing potential participants could have affected the representativeness of the sample. Therefore, respondents may have been the most engaged PHNs in healthcare for children with obesity, or, conversely, the most engaged PHNs could have been underrepresented if they did not have time to answer the questionnaire.

A disadvantage of the online data collection method was that we could not ask follow-up questions. For example, PHN 2 wrote, “*When do we talk about the obesity problem in relation to neglect*?” We recognize it would have been interesting to ask for a deepening of this reflection.

To promote trustworthiness in our study, we reflected on our preconceptions throughout the process, especially when analyzing the texts. During the analysis process, several colleagues supported us with their reflections through peer debriefing, which led to some changes in the themes.

The experiences shared by PHNs in this study can inspire new studies on the prevention of obesity in CHCs. According to our findings and other research on obesity prevention (Nygaard & Øen, 2024; Sjøvold & Øen, 2023; Westergren et al., 2021), more research should be conducted to investigate PHNs’ use of evidence-based practice and guidelines, clinical judgment in the assessment of obesity in young children, and factors that affect the implementation of national guidelines. An observational study conducted in CHCs could provide useful insight into (a) both PHNs’ and families’ actions and understandings of obesity prevention, (b) the cooperation between disciplines, municipal health services, and specialist health services, and (c) the cooperation between health providers and child welfare services in cases of obesity.

## Conclusion

PHNs strive to support overweight children and their families and feel a strong sense of responsibility for the prevention and follow-up of overweight and obesity among children. The national guidelines are seen as valuable, but too wide-ranging. BMI curves are helpful for assessing weight categories and supporting communication with parents, but parents often question this information.

PHNs found conversations about weight difficult but acknowledged the need to build mutual respect and understanding for parents to benefit from follow-ups and be motivated to accept help. Challenges included insufficient time to build productive relationships and offer follow-ups, insufficient MI skills, and inadequate support from GPs and management. Most of the barriers were at the organizational level, with a systematic failure to prioritize obesity prevention in the community. The PHNs called for increased employer support and skills development in assessing children’s weight status and conducting MIs, better organized interdisciplinary collaboration, and more resources to support their work with parents. It was suggested that The Norwegian Directorate of Health needs to expand national guidelines and provide better guidance on parent interaction.

This study provides new insights into PHNs’ experiences in identifying children’s weigh issues, parent communication, and follow-up. Furthermore, our results could be used by healthcare professionals, National Health Service management, and/or politicians, to improve the quality of childhood obesity prevention services.
